# A multi-country study of the economic burden of dengue fever: Vietnam, Thailand, and Colombia

**DOI:** 10.1371/journal.pntd.0006037

**Published:** 2017-10-30

**Authors:** Jung-Seok Lee, Vittal Mogasale, Jacqueline K. Lim, Mabel Carabali, Kang-Sung Lee, Chukiat Sirivichayakul, Duc Anh Dang, Diana Cristina Palencia-Florez, Thi Hien Anh Nguyen, Arthorn Riewpaiboon, Pornthep Chanthavanich, Luis Villar, Brian A. Maskery, Andrew Farlow

**Affiliations:** 1 Department of Zoology, University of Oxford, Oxford, United Kingdom; 2 International Vaccine Institute, Seoul, South Korea; 3 Department of Tropical Pediatrics, Faculty of Tropical Medicine, Mahidol University, Bangkok, Thailand; 4 National Institute of Hygiene and Epidemiology, Hanoi, Vietnam; 5 Clinical Epidemiology Unit, School of Medicine, Universidad Industrial de Santander, Bucaramanga, Colombia; 6 Department of Pharmacy, Mahidol University, Bangkok, Thailand; Institute for Disease Modeling, UNITED STATES

## Abstract

**Background:**

Dengue fever is a major public health concern in many parts of the tropics and subtropics. The first dengue vaccine has already been licensed in six countries. Given the growing interests in the effective use of the vaccine, it is critical to understand the economic burden of dengue fever to guide decision-makers in setting health policy priorities.

**Methods/Principal findings:**

A standardized cost-of-illness study was conducted in three dengue endemic countries: Vietnam, Thailand, and Colombia. In order to capture all costs during the entire period of illness, patients were tested with rapid diagnostic tests on the first day of their clinical visits, and multiple interviews were scheduled until the patients recovered from the current illness. Various cost items were collected such as direct medical and non-medical costs, indirect costs, and non-out-of-pocket costs. In addition, socio-economic factors affecting disease severity were also identified by adopting a logit model. We found that total cost per episode ranges from $141 to $385 for inpatient and from $40 to $158 outpatient, with Colombia having the highest and Thailand having the lowest. The percentage of the private economic burden of dengue fever was highest in the low-income group and lowest in the high-income group. The logit analyses showed that early treatment, higher education, and better knowledge of dengue disease would reduce the probability of developing more severe illness.

**Conclusions/Significance:**

The cost of dengue fever is substantial in the three dengue endemic countries. Our study findings can be used to consider accelerated introduction of vaccines into the public and private sector programs and prioritize alternative health interventions among competing health problems. In addition, a community would be better off by propagating the socio-economic factors identified in this study, which may prevent its members from developing severe illness in the long run.

## Author summary

Dengue fever has been prevalent in South-East Asia and South America. Despite the increase of dengue fever cases, there continues to be a lack of economic assessment partly due to the absence of vaccines until recent times. Many of the previous economic burden studies for dengue fever were not standardized, making them difficult to compare. We implemented the standardized economic burden survey for dengue fever in a multi-country setting: Vietnam, Thailand, and Colombia. We found that the economic burden of dengue fever is substantial in all three dengue endemic countries. Our study also identified socio-economic factors which are related to the probability of experiencing severe illness. The first live attenuated, tetravalent dengue vaccine (CYD-TDV) has been already licensed in some dengue-endemic countries. As three countries will soon face decisions on whether and how to incorporate current and future vaccine candidates within their budget constraints, the updated economic burden estimates can be used to develop sustainable financing plans.

## Introduction

Dengue fever is a major public health concern in many parts of the tropics and subtropics. Dengue is a vector-borne viral illness and transmitted to humans by two mosquito vectors: *Aedes aegypti* and *Aedes albopictus*. There are four serotypes that cause dengue, with a wide clinical spectrum of symptoms. A previous study estimates there are 96 million apparent and 294 million inapparent dengue infections occurring yearly [1].

While many countries in the tropics and subtropics recognize dengue as a public health concern, dengue control efforts were not often considered as a priority due to the absence of specific treatment and costly vector control interventions in developing countries [2]. Having reviewed publicly available literature on the economic burden of dengue, there were a relatively small number of empirical studies. While Suaya et al. covered eight countries with a common method, many of the other existing studies were not standardized in terms of their methodologies, with varying assumptions making them difficult to compare [3–5].

Given that there continues to be a lack of economic assessment of dengue, evidence on the economic burden of dengue fever is urgently needed to guide decision-makers on the expected returns on their investments and to set health policy priorities [3]. The Dengue Vaccine Initiative (DVI) has conducted extensive multidisciplinary dengue-fever studies for decision-makers in three countries: Vietnam, Thailand, and Colombia [6]. The rationale for these studies is to contribute data to inform policymakers about the economic burden of dengue fever and to assess the economic efficiency of vaccine introduction strategies.

In Vietnam, dengue is highly prevalent, and the incidence rate is reported to be 145/100,000 population according to the national surveillance system data from 2010 [7,8]. All four dengue serotypes circulate [9]. Vietnam seems to follow similar trends to Thailand where dengue epidemiology has been studied extensively. Despite mosquito control efforts, dengue fever/dengue hemorrhagic fever (DF/DHF) has steadily increased in both incidence and range of distribution in Thailand, and the incidence rate in 2010 was reported to be 177/100,000 population [8,10]. Dengue has now spread to all provinces, districts, sub-districts and communities, and is seen every year [11].

Meanwhile dengue has been prevalent for the past 20 years in Colombia. In 2010, Colombia experienced a nationwide epidemic with a dengue incidence rate of 685/100,000 population [8,12]. Further, outbreaks have been observed in many parts of the country since 2011. Dengue epidemiology in Colombia is characterized by the circulation of all four dengue serotypes. The country has a well-established national dengue surveillance system.

A recombinant tetravalent vaccine candidate (CYD-TDV) has completed Phase 3 clinical trials in Southeast Asia and South America, and has been already licensed in some dengue-endemic countries. Understanding the benefits of vaccination is necessary in order to facilitate accelerated development and introduction of safe and effective dengue vaccines into public-sector programs of dengue-endemic countries.

These three endemic countries will soon face decisions on whether, and how, to incorporate current and future vaccine candidates within their highly budget-constrained national vaccination programs [8]. As population-wide vaccination campaigns must be carefully determined considering the allocation of scarce resources among competing health problems [13], the updated economic burden estimates of dengue from this study can be used to develop cost-effective vaccination strategies and sustainable financing plans for dengue vaccine introduction in Vietnam, Thailand, and Colombia. Furthermore, taking advantage of the standardized study design in a multi-country setting, this study attempts to explore and understand socio-economic factors affecting the level of disease severity that results in different levels of economic burden.

## Methods

### Study design

**Fig 1:**
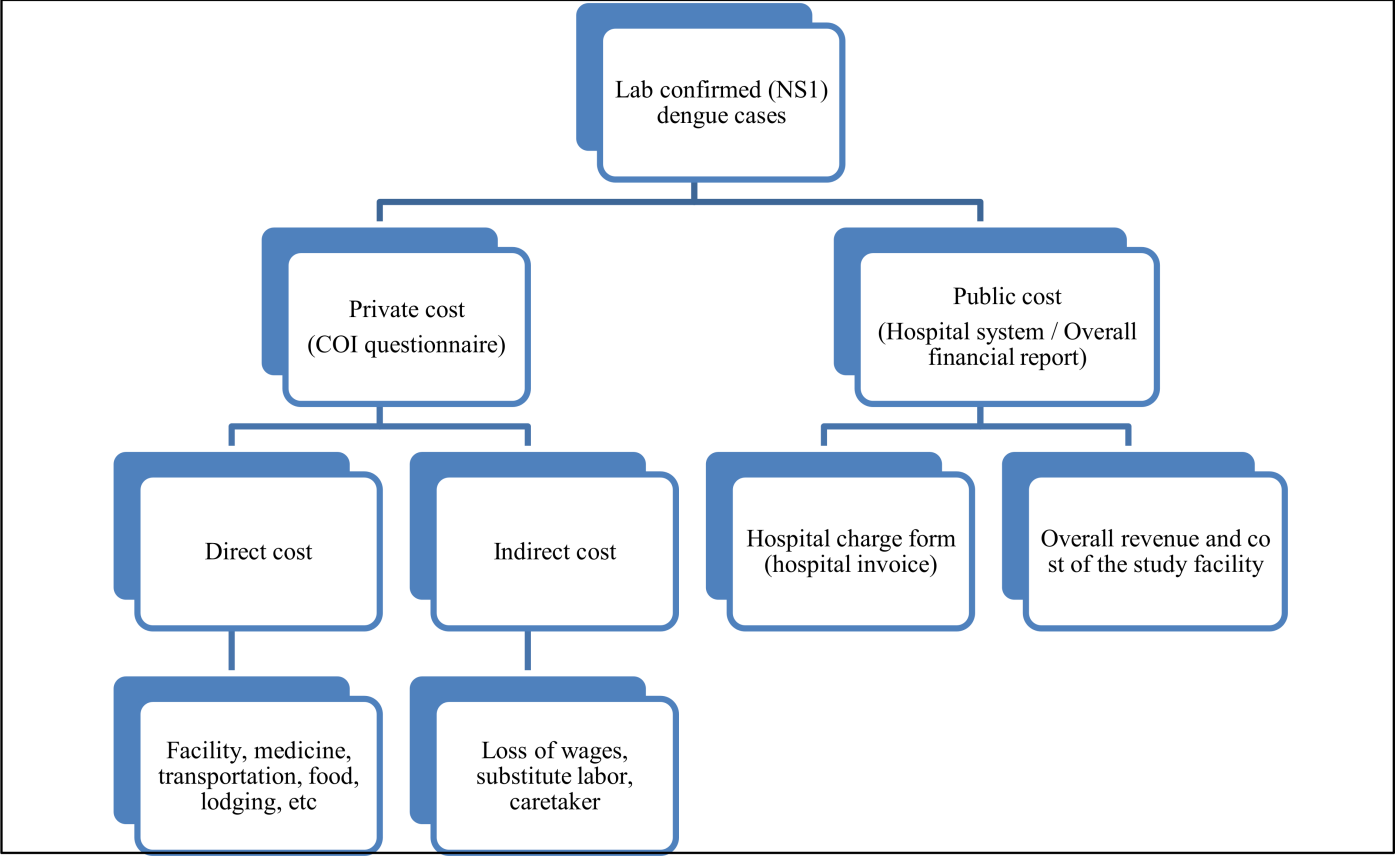
Overall study design.

Table 1 summarizes the study areas for three countries, and the overall study design is shown in Fig 1. The economic burden survey was conducted in locations where DVI’s epidemiologic studies were being carried out at the same time. The health facilities were chosen because they were main hospitals and centers providing care for the defined catchment area population. In Vietnam, Khan Hoa General Hospital is a tertiary care facility with 1,000 beds and the largest hospital in the province. The hospital is staffed with 195 doctors, 59 assistant doctors, 5 pharmacists, and 336 nurses. Bang Phae Community Hospital (BPCH) is a primary healthcare provider in Bang Phae district in Thailand. The facility is a 48-bed medium-sized tertiary care facility which conducts up to 400 outpatient consultations per day. Two health facilities were chosen in Colombia. Clinic of Piedecuesta is a secondary and tertiary care facility with a total of 42 beds. The clinic has 19 general practitioners, 36 specialists and assistants, and 39 nurses. Hospital de Piedecuesta is a 27-bed medium sized primary care facility that conducts up to 76.9% of its consultations for outpatients, with the rest being for the emergency service. Among patients who came to one of the study facilities listed in Table 1, febrile patients experiencing fever for less than 7 days were recruited for rapid tests. The economic burden survey was administered for those who were positive on the test result and who consented to participate in the survey. There were three sections in the cost-of-illness (COI) survey: day 1, day 10~14, and day 28. As part of the study procedure, the febrile patients were tested on the first day of their clinic visit, and COI surveys were administered to patients throughout the duration of their illness. On day 1, dengue-confirmed patients were asked whether they had visited any health facilities prior to the current visit (visit 1). If they had made any visits, interviewers asked how much money they had paid for direct medical and direct non-medical costs such as facility fees, medications, food, lodgings, and transportation, etc. Patients were reminded to come back to the hospital in 10 to 14 days, and, so as to prevent recall bias, they were provided with a diary card to record costs for further treatments, and other indirect cost information, such as substitute labor or caregivers. On the second visit (day 10 to 14), direct medical and non-medical costs spent during the first visit and any additional visits before the second interview were collected. In addition, patients were asked if any indirect costs had occurred such as wage loss, costs for substitute labor and/or caretakers. Once direct and indirect cost information was obtained, interviewers asked patients about socio-economic questions such as patients’ household income, education, general perceptions towards dengue fever, and routine vector control activities, etc. If there were any patients who were still feeling sick at the end of their second visit, a third interview was scheduled for day 28. The third questionnaire covered the period between day 10–14 and day 28. The day 28 survey could be conducted via telephone rather than in-person if necessary. The project-related tests were omitted from the analyses, as these may not have been incurred under routine clinical practice.

**Table 1:** Study areas.

Country	Province	City (or district)	Study period	Health facility for study
Vietnam	Khanh Hoa	Nha Trang	Oct, 2011—Oct, 2012	Khan Hoa General Hospital
Thailand	Ratchaburi	Bang Phae	Oct, 2011—Dec, 2016	Bang Phae Community Hospital
Colombia	Santander	Piedecuesta	Aug, 2014—Jul, 2015	Hospital de Piedecuesta, Clinica Piedecuesta

### Direct cost

Direct cost can be divided into two categories: (1) direct medical costs; and (2) direct non-medical costs. Direct medical costs include consultation, medication, and laboratory test costs. Patients’ out-of-pocket costs for these categories were first assessed through a questionnaire. Respondents were asked whether total treatment cost was covered by the patient (or his/her family members), insurance (private, public), or both. Because payments made by patients may not always cover the entire costs of medical treatments, hospital bill records for patients who were enrolled in the study were accessed to identify treatment costs that were not captured by patients’ out-of-pocket costs. In Colombia and Thailand, the hospital records were computerized and showed different sources of payments such as from private / public insurance, etc. However, no such system was available in Vietnam, thus medical service utilization forms, which recorded the type of services provided and hospital charges for each service, were employed for enrolled dengue fever patients in Vietnam. The data from the private surveys was then linked to the treatment records of patients (hospital charges) to better understand the full spectrum of the costs of dengue and how the economic burden is distributed among the public and private sectors.

#### Ratio of cost-to-charge

While some studies report patients’ out-of-pocket costs–which are part or all of hospital charges–as direct medical costs of dengue fever [14,15], adjusting hospital charges by the cost-to-charge ratio is another way to understand the overall societal cost of a disease. The cost-to-charge ratio is estimated by dividing the total annual hospital costs by the total annual revenue of the hospital [16]. The rationale behind the use of the cost-to-charge ratio is that in many circumstances, for various reasons, costs may not be the same as charges [17]. Charges can be higher than costs because charges may include a component to cover replacement and expansion of facilities or the need to cover losses from those who cannot afford services. In some contexts, hospital charges are lower than costs because health facilities are subsidized by governments or non-governmental organizations (NGOs) in rural areas of less-developed countries where not many people can afford health services. While the overall societal cost of a disease can be estimated by multiplying hospital charges by the cost-to-charge ratio, the direct economic impact on patients to be treated for that disease can be better assessed by the overall hospital charge information [17]. In this study, the ratios of cost-to-charge (RCC) were separately estimated for all three sites. In Vietnam and Thailand, one of the study team members was assigned to access the financial information of the study facilities. One of the study facilities in Colombia produces a comprehensive financial report every year which consists of hospital revenue from various sources and all costs including staff salary and capital assets, etc. However, such information was not obtained for the other health facility in Colombia due to logistical issues. While the financial structure of health facilities could vary, the study team ensured it included the following components: (1) the costs covered labor (i.e. staff salary, welfare), material and capital costs occurring from all sources. Capital costs include equipment, vehicle, building, and all other construction after taking into account depreciation. (2) the charges included hospital revenue from all services provided and external funding. Both overall charges and societal costs are presented.

Direct non-medical cost covers the expenses related to transportation, food, and lodgings incurred by a patient when seeking treatment, as well as by individuals accompanying a patient. The respondents were asked to provide direct medical and non-medical cost information for all visits that a patient made during their illness.

### Indirect cost

Indirect cost consists of three components: patient wage loss due to illness; substitute labor cost; and caretaker’s cost.

#### Patient productivity loss

A patient or his/her guardian was asked what the patient would have been doing if the patient had not been sick and how much the patient normally gets paid for one day’s work if the patient earns wages. These questions were followed by determining the total number of days the patient lost wages due to illness. For patients who make any income, their productivity loss was calculated by multiplying the self-reported daily wage by the total number of wage-loss days. In addition, a series of questions was asked to determine the number of days that the patient was completely unable to perform, partially unable to perform, and completely able to perform his/her usual activities. For those who do not earn any income, a full-day loss was considered for the days that they were completely unable to perform, and a half-day loss was assigned for the days that they were partially unable to perform their usual activities. In order to estimate a student’s loss in monetary value, the number of the student-loss days was multiplied by the government expenditure per primary student expressed in USD [18]. The government expenditure per primary student includes general government expenditure such as current, capital, and transfers for a given level of education. While this indicator is useful for comparing average spending on one student between countries, the estimates would be conservative because the indicator does not reflect total spending per student, including household contributions. For patients who normally perform housework and did not earn any wage, their productivity loss was converted into monetary value using the minimum wage for that country (VND 87,500 per day in Vietnam, THB 300 per day in Thailand, and COP 25,667 per day in Colombia) [19–21].

#### Substitute labor costs

Some patients need to hire substitute labor in order to carry out his/her work as the patient is unable to perform usual activities due to illness. Substitute labor might be a member of the household or someone who is hired externally. After identifying the number of substitute laborers and the relationship of the substitute laborer to the patient, respondents were asked whether they were paid or not. If paid, the daily payment for hiring a substitute laborer was recorded. Opportunity costs were also assessed. The opportunity cost of a choice is the loss of potential gain from other alternatives by choosing that specific activity. The number of days that a substitute laborer had to cut back on his/her usual activity was collected and multiplied by a daily payment for substitute laborer’s original activity to calculate the total opportunity cost. If the substitute laborer was compensated in lieu of his/her usual activities, only the amount of money that was paid for the entire duration of the service was counted. If the substitute laborer was not paid for doing the patient’s work and had to cut back on his/her usual activity, then the opportunity cost was added to the substitute labor cost. Care should be taken when considering patient wage loss and substitute labor costs because one may think that a patient’s work-day loss would be compensated by substitute labor. However, the way that the questionnaire was designed was such that respondents were first asked whether patients lost any wages due to illness and then they were asked questions about the substitute labor cost section. In other words, patients who did not lose any wages by hiring substitute laborer(s) would not report any wage loss in the first part. If patients reported some wage loss even if they had a substitute laborer, this should be interpreted as wage loss that the substitute laborer was not able to cover.

#### Caregiver’s costs

Some patients need to have a caregiver while they convalesce during a severe illness period. Opportunity cost plays an important role here because most caregivers tend to be family members who do not get paid for taking care of the patient but may need to cut back on their usual activities. For these patients, the same types of questions for substitute labor were asked, and total costs for caregivers were derived in the similar way taking into account the opportunity costs.

The total indirect costs were estimated by summing all three components: patient wage loss; substitute labor costs; and caretakers’ costs. All local currency units were converted using the official exchange rate from World Bank and expressed in 2014 USD, and the cost outcomes were also expressed in purchasing power parity (PPP) rates in the supplementary section. The official exchange rate is the rate determined in the legally sanctioned exchange market, and the PPP exchange rate reflects that the currency of one country would be converted into another currency to buy the same amount of goods and services. The PPP rates would be useful for the international comparison of non-tradable goods. Confidence intervals were estimated using the bootstrap approach, a common non-parametric technique which deals with skewed cost data [22–24]. Bootstrapping treats the observed sample as an empirical distribution, and draws a sample repeatedly with replacement [25]. From these data, the standard error (SE) can be calculated as follows:

$$\hat{se}={\left\{\frac{1}{k-1}\sum {\left(\hat{y}-\bar{y}\right)}^{2}\right\}}^{\frac{1}{2}}$$

where $\hat{y}$ is the statistic for the i^th^ bootstrap sample, $\bar{y}$ is the value of the statistic calculated using the original observations, and k is the number of replications [25]. The percentile method (2.5^th^ and 97.5^th^ percentiles of the distribution) was used to produce a 95% confidence interval [23,25].

### Disease severity

While dengue infection usually causes flu-like illness with mild symptoms, the infection occasionally develops into a potentially lethal complication called severe dengue [26]. Severe dengue has become a leading cause of hospitalization and death in some Asian and Latin American countries.

The DVI’s cost-of-illness (COI) surveys include questions not only on direct and indirect cost information of dengue fever, but also on general socio-economic information such as education, routine vector control activities, and general perceptions towards dengue fever. Questions were identically administered at all three sites, and these variables can be compared by disease severity. In this study, severe illness was defined if a patient was either hospitalized or confirmed as having dengue hemorrhagic fever (DHF) based on clinical symptoms. Whether a patient goes through severe illness or not is a dichotomy, and a dichotomous dependent variable can be considered as a function of healthcare-seeking behavior, education, and household perceptions on dengue fever and likelihood, etc. A logit model is suitable for this analysis because such models handle categorical dependent variables using maximum likelihood estimation. The binomial logistic regression estimates the log odds that individuals will be in each of two categories of a dichotomous dependent variable.

$$\ln\;\left(\frac{\pi }{1-\pi }\right)=\overset{K}{\underset{k=0}{\sum }}x_{k}\beta_{k}$$

where K is the number of independent variables, x_ik_, β are the coefficients, and the dependent variable is the log of the expected probabilities of being in each of the two categories, conditional on the values of the independent variables. Given that the surveys were standardized across the three sites, all samples were combined to achieve a larger sample size for robust model outcomes.

### Ethics statement

The cost-of-illness survey questionnaires were approved by the ethical review committees in three countries (National Institute of Hygiene and Epidemiology in Vietnam, Faculty of Tropical Medicine, Mahidol University in Thailand, Universidad de Santander in Colombia), as well as Ministry of Health in three countries and Institutional Review Board of the International Vaccine Institute. Written informed consent was obtained prior to conducting interviews, and respondents were informed that they could terminate interviews at any time. If any study participants were minors, their parents or guardians provided consent on behalf of all child participants under the age of 18 years old.

## Results

Table 2 shows patient characteristics for each study site by patient type (see S1 Table for additional information). The average number of total sick days ranges from 7 to 10 for inpatients and 6 to 9 for outpatients. While a majority of the patients sought treatment prior to the study enrollment in Vietnam, less than half of the patients did so in Thailand and Colombia. More patients tended to have caregivers than substitute labor in all three countries. In Vietnam, more than 80% of the patients responded that they had caregivers. On average, patients older than 5 years were not completely able to perform their usual activities for 5 to 7 days as inpatients, and 3 to 5 days as outpatients. The average age of the patients was 20–23 years in Vietnam and Colombia, and 15 years in Thailand. The self-reported mean household income per month was $423, $548, and $665 in Vietnam, Thailand, and Colombia, respectively.

**Table 2:** Descriptive statistics.

	Vietnam (SD)		Thailand (SD)		Colombia (SD)	
	Inpatient	Outpatient	Inpatient	Outpatient	Inpatient	Outpatient
N	59	92	45	40	70	160
Sick days prior to enrollment	3.7 (1.8)	2.8 (1.4)	2.7 (1.3)	2.2 (1.2)	5.1 (1.5)	4.3 (1.6)
Total sick days	7.4 (2.9)	6.2 (1.7)	7.8 (2.1)	6.1 (1.4)	10.4 (2.9)	8.7 (2.4)
% seeking treatment prior to enrollment	96.6% (0.2)	95.7% (0.2)	17.8% (0.4)	20.0% (0.4)	32.9% (0.5)	43.1% (0.5)
% patients with substitute labor	50% (0.5)	11.1% (0.3)	0.0% (0.0)	0.0% (0.0)	7.0% (0.3)	17.8% (0.4)
% patients with caretakers	98.3% (0.1)	85.9% (0.4)	46.7% (0.5)	12.5% (0.3)	40.0% (0.5)	48.8% (0.5)
No. of full days lost due to illness	6.7 (2.9)	5.4 (2.0)	4.7 (2.6)	2.5 (2.1)	5.8 (2.2)	4.7 (2.0)
No. of partial days lost due to illness	0.4 (1.1)	0.7 (1.6)	2.8 (1.7)	3.0 (2.0)	3.1 (1.5)	2.8 (1.9)
Average patient age	21.5 (12.8)	20.2 (9.3)	16.3 (9.3)	14.6 (9.7)	23.2 (12.7)	20.6 (13.1)
Average household income	$453 (304.2)	$404 (315.9)	$470 (227.1)	$635 (373.6)	$618 (456.1)	$686 (385.7)

**Fig 2:**
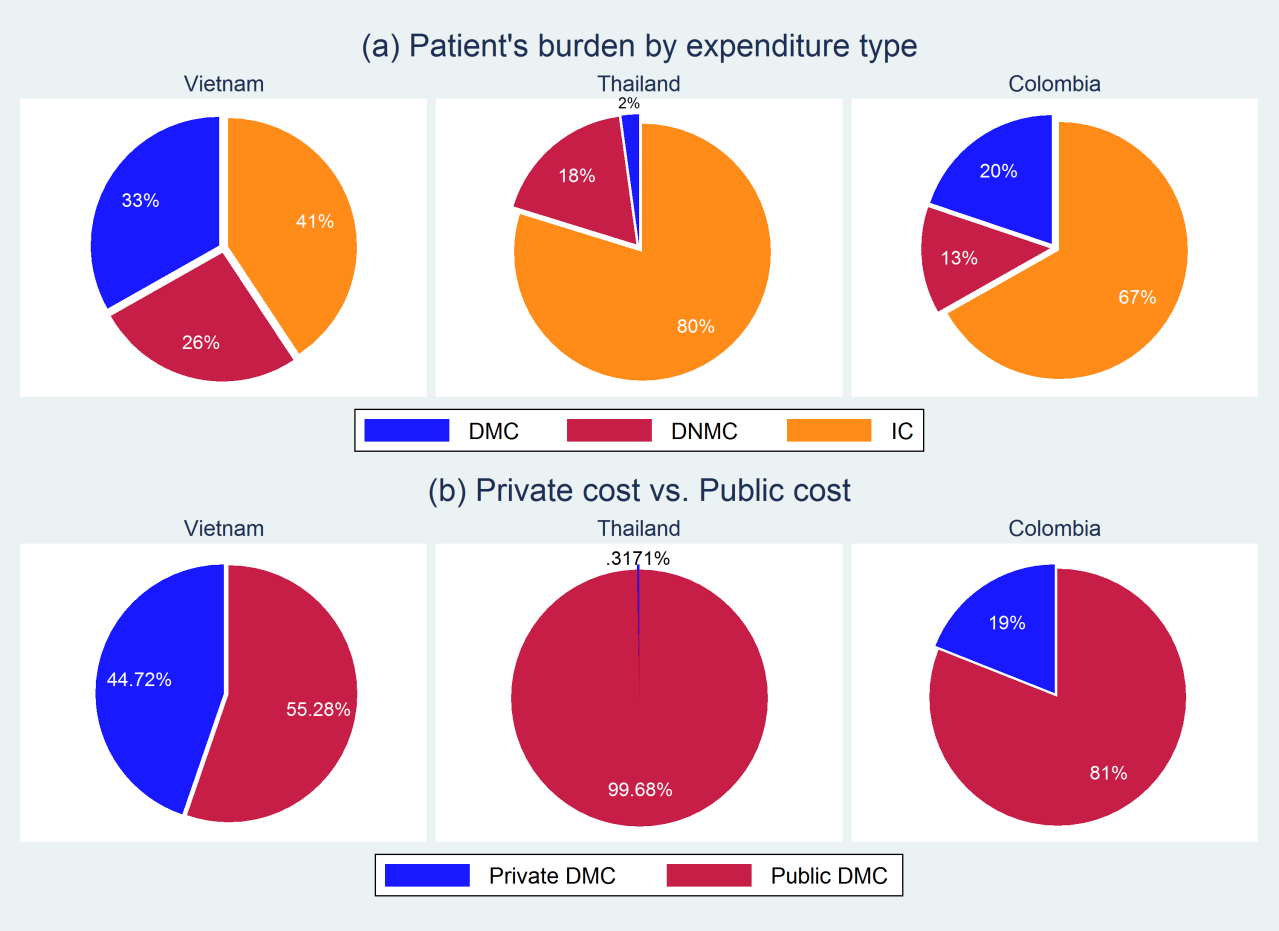
The percentage share of the economic burden for dengue fever by (a) expenditure type and (b) payer type.

Fig 2 summarizes the proportions of patient burden by expenditure, as well as the percentage share of patient’s out-of-pocket (OOP) costs versus non-OOP costs, such as government subsidy or payments from other insurance schemes. For all three countries, indirect cost accounts for the highest proportion of patient’s OOP among the three expenditure types in Fig 2(A): direct medical cost (DMC), direct non-medical cost (DNMC), and indirect cost (IC). While DMC is the second highest burden for patients in Vietnam and Colombia, DMC is only 2% in Thailand. This is mainly because people in Thailand are covered by its universal healthcare system so patients do not have to pay for most of the OOP costs related to direct medical services. This is more apparent in Fig 2(B). About 99% of DMC are covered by the public sector, which is the universal healthcare system in Thailand. The proportion of the non-OOP costs is also high in Colombia, at 81%. In Vietnam, public DMC coverage is less than that of the other two countries, meaning that more patients tend to pay directly for the medical services that they receive.

**Fig 3:**
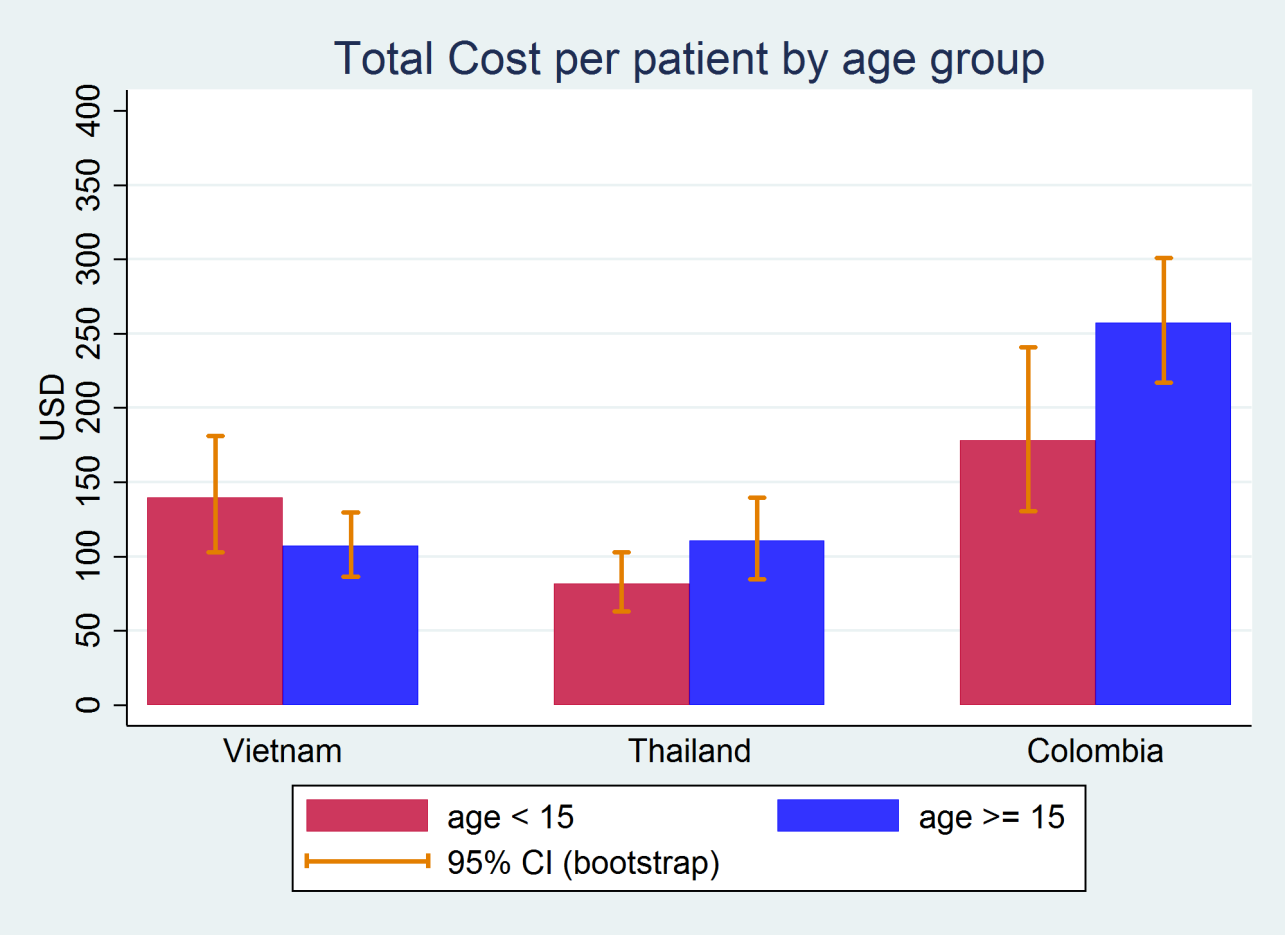
Total cost per patient by age group.

The economic burden of dengue fever is shown in Table 3. DMC is highest among the three expenditure types, after taking into account both private and public spending. Total cost per inpatient episode is $200, $141, and $385 in Vietnam, Thailand, and Colombia, respectively. In the case of outpatients, total cost per episode ranges from $40 to $158, with Colombia having the highest and Thailand having the lowest. It is interesting to see that IC and DNMC are higher in Vietnam than in Thailand despite the higher average income in Thailand. This is because there was no reported substitute laborer cost in Thailand, and the duration of caregiver’s work was shorter in Thailand than in Vietnam. In addition, while the meal service was included for inpatients in the hospital charge in the health facility of Thailand, all patients had to pay extra for food in Vietnam. Considering the mean duration of the disease, total cost per day was calculated. It appeared that Colombia had again the highest cost per day, followed by Vietnam and Thailand. The societal total costs were also estimated after adjusting for the ratio of cost-to-charge (RCC). While the total cost per episode went up after the adjustment in Vietnam and Thailand, it went down in Colombia. By age group, the total cost per episode is higher for the older age group than the younger age group in Thailand and Colombia, but this is the opposite in Vietnam as shown in Fig 3. This is mainly due to the high number of inpatients than outpatients in the younger age group in Vietnam. The economic burden was further disaggregated by age group, patient type, and cost type in Table 4.

**Table 3:** Average economic burden of dengue fever per episode^a^.

	Vietnam^b^						Thailand						Colombia					
	Inpatient (n = 59)			Outpatient (n = 92)			Inpatient (n = 45)			Outpatient (n = 40)			Inpatient (n = 70)			Outpatient (n = 160)		
	USD^c^	BT CI^c^ (lower, upper)		USD	BT CI (lower, upper)		USD	BT CI (lower, upper)		USD	BT CI (lower, upper)		USD	BT CI (lower, upper)		USD	BT CI (lower, upper)	
Direct Medical Cost (DMC)	$83	$62	$108	$26	$22	$30	$81	$67	$95	$7	$6	$8	$245	$199	$297	$33	$30	$36
Direct Non-Medical Cost (DNMC)	$51	$44	$58	$9	$7	$13	$13	$7	$21	$5	$4	$6	$27	$20	$34	$17	$15	$21
Indirect Cost (IC)^d^	$67	$48	$89	$27	$20	$36	$47	$36	$59	$28	$23	$33	$113	$85	$144	$107	$76	$145
Total Cost	$200	$165	$239	$62	$52	$74	$141	$119	$164	$40	$34	$45	$385	$331	$443	$158	$124	$197
Total Cost per Day	$29	$24	$34	$10	$8	$12	$19	$16	$22	$7	$6	$7	$38	$32	$44	$18	$14	$22
Total Cost (RCC adjustment)	$213	$175	$256	$64	$54	$76	$181	$153	$210	$43	$37	$48	$278	$239	$318	$144	$111	$183

^a^ All local currency values were converted using the official exchange rates from World Bank. See S2 Table for values converted using the Purchasing Power Parity (PPP) conversion factor

^b^ Because data collection was done in 2012 in Vietnam, the estimates were inflated to 2014 USD.

^c^ Bootstrapping with the percentile method.

^d^ See S3 Table for further break-down of indirect cost

**Table 4:** Disaggregated average economic burden of dengue fever per episode^a^.

	Vietnam						Thailand						Colombia					
	Inpatient			Outpatient			Inpatient			Outpatient			Inpatient			Outpatient		
	USD	BT CI (lower, upper)		USD	BT CI (lower, upper)		USD	BT CI (lower, upper)		USD	BT CI (lower, upper)		USD	BT CI (lower, upper)		USD	BT CI (lower, upper)	
Direct Medical Cost (DMC), age < 15	$106	$61	$170	$24	$17	$31	$73	$53	$93	$6	$5	$8	$212	$149	$283	$31	$26	$36
Direct Medical Cost (DMC), age ≥ 15	$72	$52	$97	$27	$22	$32	$89	$70	$106	$8	$6	$11	$262	$201	$331	$35	$30	$40
Direct Non-Medical Cost (DNMC), age < 15	$55	$45	$66	$14	$7	$22	$8	$5	$11	$5	$4	$6	$33	$21	$49	$16	$12	$21
Direct Non-Medical Cost (DNMC), age ≥ 15	$49	$40	$58	$8	$5	$11	$17	$7	$33	$5	$3	$7	$23	$16	$33	$18	$15	$23
Indirect Cost (IC), age < 15	$75	$47	$111	$30	$20	$40	$52	$35	$72	$32	$26	$37	$59	$28	$98	$87	$40	$156
Indirect Cost (IC), age ≥ 15	$63	$40	$91	$26	$17	$38	$43	$29	$58	$20	$10	$29	$140	$103	$181	$121	$84	$165

^a^ All local currency values were converted using the official exchange rates from World Bank. See S2 Table for values converted using the Purchasing Power Parity (PPP) conversion factor

**Fig 4:**
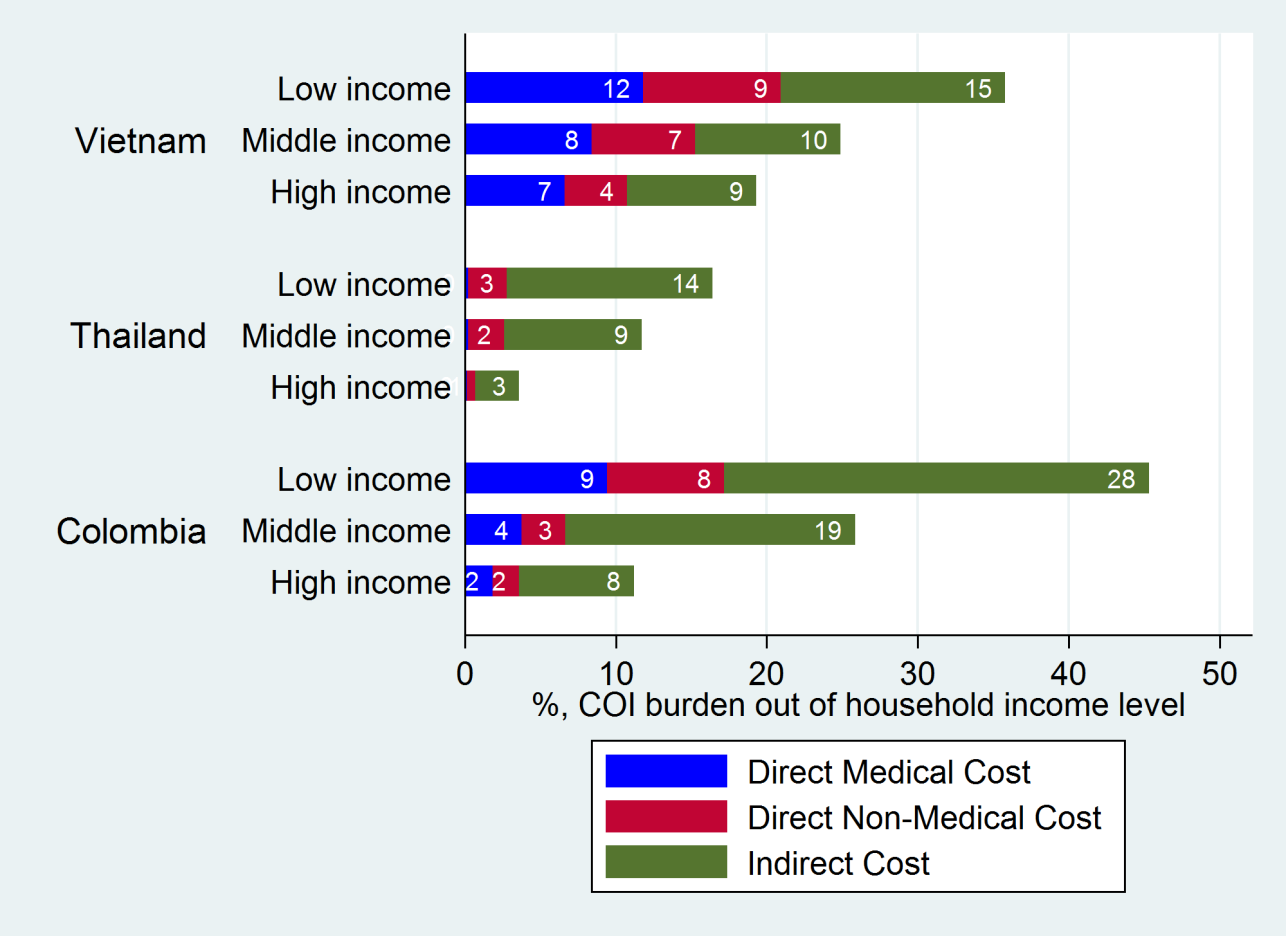
Proportion of the economic burden (OOP costs) by income group.

The economic burden of dengue fever would have varying impacts on household equity. For those who reported their monthly income, three income groups were first generated based on percentiles of monthly income: low-income group (income ≤ 25%), middle-income group (25% < income ≤ 75%), and high-income group (income > 75%). The list of household assets in the survey was designed to include various items from essentials to luxury goods reflecting the local context of each country. Given this, the level of the household assets in each income group was identified, and the respondents who did not report their monthly income were categorized into the three income groups based upon their levels of household assets. The mean income value of the group to which these households belonged was assigned. The total OOP for the economic burden of dengue fever was then estimated as a proportion of the self-reported household monthly income by income group. As shown in Fig 4, the percentage of the economic burden of dengue for the low-income group was 36%, 17%, and 45% in Vietnam, Thailand, and Colombia, respectively. The percentage of the economic burden of dengue fever decreased moving towards the higher income groups. Due to the universal healthcare system, the percentage of the DMC burden was less than 1% for all three income groups in Thailand, which reduces the overall OOP burden for patients.

Table 5 shows the regression outputs. While the type 1 model is parsimonious and only includes the variables related to the current illness, the type 2 model was run with all possible covariates including socio-economic variables. As expected, the number of sick days before and after the study enrollment was highly significant and positively related to severe illness. The seeking-early-treatment variable is also statistically significant at the 1% level and decreased the odds of experiencing severe illness for both models. This indicates that patients who sought treatment at the early stage of illness would be less likely to develop severe illness later on. While patients with severe illness tended to have more substitute labor or caretaker(s) than patients with non-severe illness, this variable is marginally significant in type 2. Compared with patients with no education, patients with some or higher education were less likely to have severe illness for the current episode. Age, household income levels, and vector control activities were not statistically significant. In addition, the dengue perception score was developed based upon respondents’ general knowledge of dengue fever, and was categorized into three levels with the lowest score indicating the poor level of knowledge on dengue fever. Having less awareness or lacking general knowledge of dengue fever was positively associated with experiencing severe illness. It was also shown that patients were less likely to develop less severe illness when there were more household members who had been previously infected and recovered. Overall, the type 2 model was preferred over the type 1 model based on the difference of the Akiake Information Criterion (AIC) values [27]. It should be noted that some of the milder cases may never receive medical attention, and this may be related to income and education levels. While this would be difficult to be statistically tested in this study given the absence of healthcare-seeking behavior indicators outside the study facilities, the distributions of non-severe and severe patients appear to be similar over income and education levels, holding other factors constant (see S1 Fig). This partly indicates that there was no serious selection bias in the dataset.

**Table 5:** Socio-economic factors influencing disease severity (regression outputs).

Independent variable^b^	Type 1			Type 2		
	Coeff (in OR^a^)	(SE)		Coeff (in OR)	(SE)	
Age (year)	1.00	(0.01)		1.01	(0.01)	
Sick days before the study enrollment	1.26	(0.08)	***	1.35	(0.09)	***
Sick days after the study enrollment	1.21	(0.05)	***	1.20	(0.05)	***
Seeking early treatment	0.55	(0.12)	***	0.44	(0.1)	***
Substitute labor/caretaker	1.34	(0.29)		1.59	(0.37)	**
Education 2 (some education)				0.35	(0.17)	**
Education 3 (university / post graduate)				0.29	(0.15)	**
Income level (middle)				0.80	(0.19)	
Income level (high)				0.96	(0.29)	
Dengue perception (poor)				1.96	(0.75)	*
Dengue perception (medium)				2.18	(0.55)	***
Previous infections within household				0.48	(0.12)	***
Vector control activity				0.93	(0.2)	
Constant	0.10	(0.04)	***	0.16	(0.1)	***
N	466.00			466.00		
Log likelihood	-290.57			-277.80		
AIC	593.13			583.59		

^a^ Odds ratio

^b^ Reference categories of the categorical variables: Education 1 (no education), Income level (high), Dengue perception (high)

* Significance at the 10% level

** at the 5% level

*** at the 1% level

## Discussion

Estimates of the economic burden of dengue fever may vary depending upon a country’s healthcare system and the different methodologies applied. While there were existing economic burden studies, the advantages of the current study are that it relies on the implementation of a standardized survey tool and the inclusion of various expenditure types and age groups in multiple countries. This makes it possible to make a more complete comparison across the three dengue-endemic countries. Using the standardized methodology, this study found that the total cost per dengue episode is $200, $141, $385 for inpatients and $62, $40, $158 for outpatients in Vietnam, Thailand, and Colombia, respectively. Among the various OOP expenditure components, indirect cost was the biggest private burden for patients in all three countries. Non-OOP payments, such as from insurance or nationwide healthcare system, reduce patients’ burden of DMC. The patients’ share of burden of DMC was the least in Thailand, followed by Colombia and Vietnam. In all three countries, the percentage of the private economic burden of dengue fever was highest in the low-income group and lowest in the high-income group.

Though many previous studies were not based on standardized methodologies, making them difficult to compare [3,4], some existing studies that did not use secondary data sources or extrapolation were compared with the current study outcomes (see S4 Table for summary). Tam et al. reported $167.8 per DHF inpatient episode in 2006 in Vietnam [28]. Once the estimate was inflated to 2014 USD using consumer price index, this value was higher than our estimate. However, it should be noted that this previous study only looked at DHF inpatients who were supposed to experience more severe illness. Another study by Harving et al. showed the average total cost of $61.4 per DHF inpatient younger than 15 years in 2005 [29]. Our estimate per inpatient episode for the same age group is $125.2, which is less than Harving et al.’s estimate after inflation adjustment ($147.1), but again Harving et al. also looked only at DHF inpatients. In Thailand, while Anderson et al. and Clark et al. reported the average total cost of $31.8 to $44 per inpatient and $10.2 per outpatient [14,30], Okanurak et al. estimated the total cost per inpatient as $172.9 and $141.5 from two different sites in Thailand [31]. The estimates from Anderson et al. and Clark et al. were lower than our study estimates even after inflation adjustment. One of the main reasons is that the former two studies focused on patients’ OOP costs, whereas our study took into account both patients’ OOP payments and public payments, which would make for a large difference especially in the context of Thailand. On the other hand, Okanurak et al. looked at DHF patients, resulting in higher costs than our study estimates. Suaya et al. also estimated dengue cost of illness in Thailand and reported $573 for inpatients, which is a lot higher than the other study estimates including ours [5]. Rodriguez et al. estimated a total cost of $497.9 per inpatient and $202.3 per outpatient in Colombia. There are similarities between this previous study and our study in terms of the cost items considered and the sub-component estimates. They reported higher indirect cost estimates, making the overall costs higher than our estimates. This could be partly because the previous study included projected lost income due to death, whereas the current study did not have any deaths occurred during the study period.

In addition to the estimation of the economic burden of dengue fever, the logit model was constructed to identify factors explaining variance of disease severity. The logit analyses showed that early treatment, higher education, and better knowledge of dengue disease would be associated with a reduction of the probability of developing more severe illness. This can be partly explained by the fact that secondary dengue infection which would likely cause more severe illness would be inversely correlated with the independent variables. As the disease ranges from flu-like mild symptoms (dengue) to potentially lethal complications (severe dengue), resulting in different levels of economic burden, a community would be better off expanding the factors identified in this study, which may prevent its members from developing severe illness in the long run.

Some areas of uncertainty deserve attention. While the study captured all patients’ OOP costs spent during the entire period of illness by conducting multiple interviews, public DMC payments and RCCs were limited to our study’s health facilities. Because a majority of the costs were incurred during the enrollment visit at the study’s facilities rather than at referral facilities or pharmacies across the study sites, the estimates would not be greatly influenced. However, our estimates are conservative. In Colombia, where two health facilities were involved, the RCC was only obtained from one of the facilities due to logistical issues. Because these study facilities were in the same district, a single RCC was used for all patients. While various cost and charge items were taken into account for the estimation of the RCC, the detailed list of financial information was confidential for some of the health facilities, limiting the way that the estimates were presented. Ideally, our study samples should be more heterogeneous and representative of the entire countries. Given that this study was carried out in locations where epidemiologic studies were being conducted at the same time, care must be taken when generalizing the estimates beyond the study’s communities. Nonetheless, hospital charges may vary in different locations of a country, but within the same country the unit prices for service items may not be as variable as the charges. Assuming the duration of illness is similar within a country, factors which may influence the total cost adjusted by the ratio of cost-to-charge in the same country are patient’s out-of-pocket expenditures for direct non-medical costs and indirect costs. Because these are directly related to the income level in a community, it would be possible to understand the representativeness of our estimates by comparing the average income of the study communities with Gross National Income (GNI) per capita. The study refusal rates among eligible patients were 2.3% and 1.4% in Thailand and Colombia respectively. Due to logistical issues, the refusal rate could not be estimated in Vietnam. While there were no patients who felt sick after the 2nd interview of the current study, Tiga et al. reported that some dengue patients experience persistent symptoms such as asthenia, fatigue, and trouble working [32]. It should be noted that there may be patients who do not generally come to the professional health sector such as our study facilities [33]. While the current study captured any non-medical sector visits such as traditional healers or others before and after the study enrollment, there may have been non-enrolled dengue patients who did not come to any of our study facilities during the study period, and this may have been partially related to patient’s education and income levels.

The burden of dengue has been rising partly due to the absence of population-wide vaccination campaigns and the limited impacts of vector control activities. A previous study assumed that vector control would not be able to achieve permanent reduction of dengue based on the experience of Singapore [13]. The first live attenuated, tetravalent dengue vaccine (CYD-TDV) has been already licensed in some dengue-endemic countries. To maximize the effective use of a dengue vaccine, vaccination strategies should be carefully considered after taking into account various competing health problems at the country level. Identifying effective vaccination strategies requires various sources of information such as accurate incidence rates, the long-term behavior of the vaccine (i.e., waning rates of efficacy, the rate of any adverse effects, etc.) and the price of the vaccine. Along with more details, this study’s estimates of the economic burden of dengue fever can play a critical role in determining cost-effective vaccination strategies, and so facilitate the process of dengue vaccine introduction by guiding decision-makers on the expected societal health benefits through vaccination.

## Supporting information

S1 Table The number of days lost due to illness. (DOCX)

S2 Table Average economic burden of dengue fever per episode in PPP. (DOCX)

S3 Table Indirect cost break-down. (DOCX)

S4 Table Study comparisons. (DOCX)

S1 Fig The distribution of patients by education and income. (TIF)
