# Supplementary material for: A multi-country study of the economic burden of dengue fever: Vietnam, Thailand, and Colombia
Source: PLoS Negl Trop Dis. 2017 Oct 30;11(10):e0006037. doi: 10.1371/journal.pntd.0006037 (PMC5679658; doi:10.1371/journal.pntd.0006037)
Supplement: S1 Table — (DOCX) [file pntd.0006037.s001.docx]

**S1 Table. The number of days lost due to illness**

|  | **Vietnam (SD, range)** | |  | **Thailand (SD, range)** | |  | **Colombia (SD, range)** | |
| --- | --- | --- | --- | --- | --- | --- | --- | --- |
|  | **Inpatient** | **Outpatient** |  | **Inpatient** | **Outpatient** |  | **Inpatient** | **Outpatient** |
| No. of days lost (patients)^a^ | 6.9 (2.9, 0-16 ) | 5.7 (1.7, 3-10) |  | 6.1 (2.3, 2-14.5) | 4.0 (1.6, 1-8) |  | 7.4 (2.3, 3-14.5) | 6.1 (1.9, 2-13) |
| No. of days lost (substitute laborers)^b^ | 7.0 (10.1, 0-44) | 2.1 (4.0, 0-10) |  | 0.0 | 0.0 |  | 3.8 (4.3, 0-8) | 3.3 (3.7, 0-11) |
| No. of days lost (caregivers)^c^ | 8.1 (7.6, 0-44) | 2.0 (3.2, 0-14) |  | 2.1 (2.2, 0-6) | 2 (2.8, 0-6) |  | 5.4 (3.7, 0-11) | 4.9 (4.3, 0-20) |

^a^ The average number of days lost due to illness for all enrolled patients older than 5 years old.

^b^ The average number of days lost for all substitute laborers per patient among those who had at least 1 substitute laborer.

^c^ The average number of days lost for all caregivers per patient among those who had at least 1 caretaker.
