# Supplementary material for: A multi-country study of the economic burden of dengue fever: Vietnam, Thailand, and Colombia
Source: PLoS Negl Trop Dis. 2017 Oct 30;11(10):e0006037. doi: 10.1371/journal.pntd.0006037 (PMC5679658; doi:10.1371/journal.pntd.0006037)
Supplement: S2 Table — (DOCX) [file pntd.0006037.s002.docx]

**S2 Table. Average economic burden in PPP**

**Average economic burden of dengue fever per episode in PPP**

|  | **Vietnam^a^** | | | | | |  | **Thailand** | | | | | |  | **Colombia** | | | | | |
| --- | --- | --- | --- | --- | --- | --- | --- | --- | --- | --- | --- | --- | --- | --- | --- | --- | --- | --- | --- | --- |
|  | **Inpatient (n = 59)** | | | **Outpatient (n = 92)** | | |  | **Inpatient (n = 45)** | | | **Outpatient (n = 40)** | | |  | **Inpatient (n = 70)** | | | **Outpatient (n = 160)** | | |
|  | **I$^c^** | **BT CI^b^ (lower, upper)** | | **I$** | **BT CI (lower, upper)** | |  | **I$** | **BT CI (lower, upper)** | | **I$** | **BT CI (lower, upper)** | |  | **I$** | **BT CI (lower, upper)** | | **I$** | **BT CI (lower, upper)** | |
| Direct Medical Cost (DMC) | $235 | $176 | $307 | $74 | $62 | $87 |  | $214 | $178 | $250 | $18 | $15 | $21 |  | $415 | $335 | $501 | $56 | $50 | $62 |
| Direct Non-Medical Cost (DNMC) | $144 | $126 | $165 | $27 | $19 | $36 |  | $33 | $18 | $56 | $12 | $10 | $15 |  | $45 | $34 | $58 | $30 | $25 | $35 |
| Indirect Cost (IC) | $191 | $137 | $253 | $76 | $56 | $102 |  | $125 | $96 | $157 | $75 | $62 | $88 |  | $192 | $144 | $243 | $181 | $128 | $245 |
| Total Cost | $571 | $471 | $682 | $177 | $147 | $212 |  | $371 | $313 | $433 | $105 | $90 | $120 |  | $651 | $559 | $748 | $266 | $210 | $333 |
| Total Cost per Day | $82 | $67 | $98 | $28 | $24 | $33 |  | $49 | $41 | $59 | $17 | $15 | $19 |  | $64 | $55 | $74 | $30 | $24 | $37 |
| Total Cost (RCC adjustment) | $609 | $500 | $729 | $182 | $153 | $217 |  | $479 | $405 | $555 | $113 | $98 | $128 |  | $470 | $404 | $537 | $243 | $187 | $309 |

^a^ Because data collection was done in 2012 in Vietnam, the estimates were inflated to 2014 USD.

^b^ Bootstrapping with the percentile method.

^c^ All local currency values were converted using the Purchasing Power Parity (PPP) conversion factor from World Bank.

**Disaggregated average economic burden of dengue fever per episode in PPP**

|  | **Vietnam** | | | | | |  | **Thailand** | | | | | |  | **Colombia** | | | | | |
| --- | --- | --- | --- | --- | --- | --- | --- | --- | --- | --- | --- | --- | --- | --- | --- | --- | --- | --- | --- | --- |
|  | **Inpatient** | | | **Outpatient** | | |  | **Inpatient** | | | **Outpatient** | | |  | **Inpatient** | | | **Outpatient** | | |
|  | **USD** | **BT CI (lower, upper)** | | **USD** | **BT CI (lower, upper)** | |  | **USD** | **BT CI (lower, upper)** | | **USD** | **BT CI (lower, upper)** | |  | **USD** | **BT CI (lower, upper)** | | **USD** | **BT CI (lower, upper)** | |
| Direct Medical Cost (DMC), age < 15 | $301 | $174 | $485 | $67 | $48 | $89 |  | $192 | $139 | $246 | $17 | $14 | $20 |  | $358 | $252 | $477 | $52 | $44 | $61 |
| Direct Medical Cost (DMC), age ≥ 15 | $207 | $149 | $276 | $76 | $62 | $91 |  | $234 | $186 | $281 | $21 | $15 | $29 |  | $442 | $340 | $560 | $58 | $51 | $67 |
| Direct Non-Medical Cost (DNMC), age < 15 | $158 | $130 | $187 | $40 | $21 | $63 |  | $21 | $14 | $29 | $12 | $10 | $15 |  | $56 | $35 | $82 | $27 | $21 | $35 |
| Direct Non-Medical Cost (DNMC), age ≥ 15 | $138 | $115 | $164 | $22 | $14 | $32 |  | $44 | $17 | $86 | $12 | $8 | $17 |  | $40 | $27 | $56 | $31 | $25 | $38 |
| Indirect Cost (IC), age < 15 | $214 | $135 | $316 | $84 | $58 | $115 |  | $137 | $94 | $189 | $84 | $70 | $98 |  | $100 | $47 | $165 | $146 | $67 | $264 |
| Indirect Cost (IC), age ≥ 15 | $181 | $113 | $260 | $74 | $49 | $108 |  | $113 | $77 | $153 | $52 | $26 | $76 |  | $236 | $174 | $306 | $205 | $142 | $279 |
