# Supplementary material for: A multi-country study of the economic burden of dengue fever: Vietnam, Thailand, and Colombia
Source: PLoS Negl Trop Dis. 2017 Oct 30;11(10):e0006037. doi: 10.1371/journal.pntd.0006037 (PMC5679658; doi:10.1371/journal.pntd.0006037)
Supplement: S3 Table — (DOCX) [file pntd.0006037.s003.docx]

**S3 Table. Indirect cost break-down**

|  | **Vietnam** | | | | | |  | **Thailand** | | | | | |  | **Colombia** | | | | | |
| --- | --- | --- | --- | --- | --- | --- | --- | --- | --- | --- | --- | --- | --- | --- | --- | --- | --- | --- | --- | --- |
|  | **Inpatient (n = 59)** | | | **Outpatient (n = 92)** | | |  | **Inpatient (n = 45)** | | | **Outpatient (n = 40)** | | |  | **Inpatient (n = 70)** | | | **Outpatient (n = 160)** | | |
|  | **USD** | **BT CI (lower, upper)** | | **USD** | **BT CI (lower, upper)** | |  | **USD** | **BT CI (lower, upper)** | | **USD** | **BT CI (lower, upper)** | |  | **USD** | **BT CI (lower, upper)** | | **USD** | **BT CI (lower, upper)** | |
| Patient's wage loss | $15 | $9 | $22 | $18 | $13 | $24 |  | $36 | $28 | $44 | $27 | $23 | $32 |  | $91 | $66 | $119 | $47 | $37 | $59 |
| Substitute labor cost | $10 | $4 | $18 | $2 | $0 | $4 |  | $0 | $0 | $0 | $0 | $0 | $0 |  | $6 | $1 | $14 | $22 | $6 | $44 |
| Caregiver cost | $41 | $27 | $59 | $7 | $4 | $11 |  | $11 | $4 | $21 | $1 | $0 | $3 |  | $16 | $6 | $28 | $37 | $16 | $69 |

* The costs were converted by the official exchange rates

|  | **Vietnam** | | | | | |  | **Thailand** | | | | | |  | **Colombia** | | | | | |
| --- | --- | --- | --- | --- | --- | --- | --- | --- | --- | --- | --- | --- | --- | --- | --- | --- | --- | --- | --- | --- |
|  | **Inpatient (n = 59)** | | | **Outpatient (n = 92)** | | |  | **Inpatient (n = 45)** | | | **Outpatient (n = 40)** | | |  | **Inpatient (n = 70)** | | | **Outpatient (n = 160)** | | |
|  | **USD** | **BT CI (lower, upper)** | | **USD** | **BT CI (lower, upper)** | |  | **USD** | **BT CI (lower, upper)** | | **USD** | **BT CI (lower, upper)** | |  | **USD** | **BT CI (lower, upper)** | | **USD** | **BT CI (lower, upper)** | |
| Patient's wage loss | $44 | $27 | $64 | $51 | $37 | $70 |  | $95 | $74 | $116 | $72 | $61 | $84 |  | $154 | $112 | $201 | $80 | $63 | $100 |
| Substitute labor cost | $29 | $11 | $52 | $5 | $0 | $11 |  | $0 | $0 | $0 | $0 | $0 | $0 |  | $10 | $1 | $23 | $37 | $10 | $74 |
| Caregiver cost | $118 | $76 | $169 | $21 | $11 | $31 |  | $30 | $11 | $56 | $3 | $0 | $8 |  | $27 | $10 | $48 | $63 | $27 | $117 |

* The costs were converted by the PPP rates
