# Supplementary material for: A multi-country study of the economic burden of dengue fever: Vietnam, Thailand, and Colombia
Source: PLoS Negl Trop Dis. 2017 Oct 30;11(10):e0006037. doi: 10.1371/journal.pntd.0006037 (PMC5679658; doi:10.1371/journal.pntd.0006037)
Supplement: S4 Table — (DOCX) [file pntd.0006037.s004.docx]

**S4 Table. Study comparisons**

| **Country** | **Study** | **Total cost** | **Comparison with our study^a^** | **Description for differences in existing studies** |
| --- | --- | --- | --- | --- |
| Vietnam | Tam et al. | $167.80 | Higher | DHF IP only |
|  | Harving et al. | $61.40 | Higher | DHF IP only |
| Thailand | Anderson et al. | $31.8 ($10.2)^b^ | Lower | Out of pocket expenditure only |
|  | Clark et al. | $44 | Lower | Out of pocket expenditure only |
|  | Okanurak et al. | $172.9, $141.5^c^ | Higher | DHF only |
|  | Suaya et al. | $573 | Much higher | Unknown |
| Colombia | Rodiriguez et al. | $497.9 ($202.3)^b^ | Higher | Higher indirect cost accounting for death occurred |

^a^ After all adjustments as described in the text

^b^ Inpatient (outpatient)

^c^ Estimates from two different sites in Thailand
