# Supplementary figures and images for: A multi-country study of the economic burden of dengue fever: Vietnam, Thailand, and Colombia
Source: PLoS Negl Trop Dis. 2017 Oct 30;11(10):e0006037. doi: 10.1371/journal.pntd.0006037 (PMC5679658; doi:10.1371/journal.pntd.0006037)

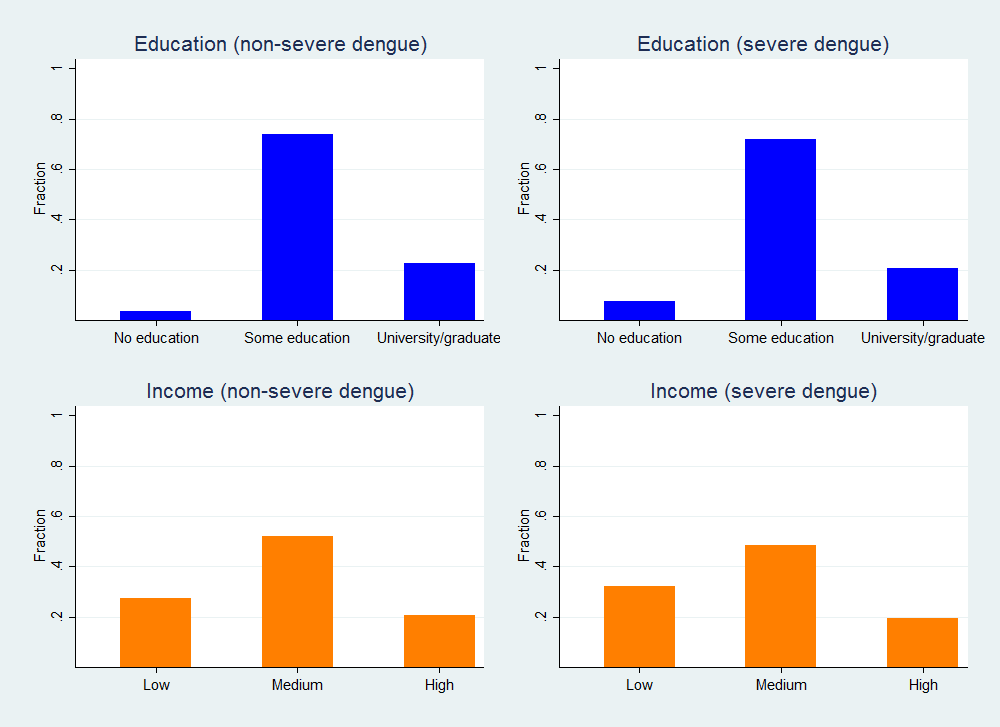

Supplement: S1 Fig — (TIF) [file pntd.0006037.s005.tif]
